# In vitro and in vivo antagonistic activity of new probiotic culture against *Clostridium difficile* and *Clostridium perfringens*

**DOI:** 10.1186/s12866-017-1015-5

**Published:** 2017-05-06

**Authors:** Nataša Golić, Katarina Veljović, Nikola Popović, Jelena Djokić, Ivana Strahinić, Igor Mrvaljević, Amarela Terzić-Vidojević

**Affiliations:** 1 Laboratory for Molecular Microbiology (LMM), Institute of Molecular Genetics and Genetic Engineering, University of Belgrade, Vojvode Stepe 444a, P. O. Box 23, Belgrade, 11010 Serbia; 2 Centre for Development and Production, Veterinary Station “Koker”, Adaševci, Serbia

**Keywords:** Probiotic, *Clostridium sp*., TGF-β, GALT, Goats

## Abstract

**Background:**

Genus *Clostridium* accompanies more than 200 known species and at least 30 among them are associated with human and animal diseases. At the moment, the treatment of clostridial infections is based on use of antibiotics. However, due to the European ban on the use of antibiotics in livestock production, novel therapeutic strategies for treatment of these hardly curable infections have been evaluated. Hence, in this study the antimicrobial effect of newly designed probiotic culture consisted of natural isolates *Lactobacillus helveticus* BGRA43, *Lactobacillus fermentum* BGHI14 and *Streptococcus thermophilus* BGVLJ1-44 against *Clostridium difficile* and *Clostridium perfringens* was analyzed.

**Results:**

The probiotic culture showed strong in vitro antimicrobial effect on *C. difficile* (human clinical isolate). In addition, individual strains and the probiotic combination exhibited immunomodulatory activity. The probiotic combination significantly increased the proliferation of GALT lymphocytes. At the other hand, none of the bacterial treatments (individual strains and the combination) induced the production of proinflammatory cytokines IL-6 and IL-1β by intestinal epithelial cells, Caco-2. Interestingly, Caco-2 cells exposed to the probiotic combination produced significantly elevated amount of TGFβ pointing to potential protecting effect of the probiotic. In addition, the results of field trial on spontaneously infected goats revealed reduction of *C. perfringens* in goats (below the detection threshold) after the probiotic treatment.

**Conclusions:**

The results of this study indicated that the novel probiotic deserves to be further investigated as a promising antimicrobial agent against *C. difficile* and *C. perfringens*.

## Background

*Clostridium difficile* and *Clostridium perfringens* are toxin-producing opportunistic pathogens potentially associated with gastrointestinal infections and allergy in humans and animals [1, 2]. *C. difficile* is the leading etiological agent of microbiota dysbiosis-associated diarrhoea caused by antibiotic use [3]. *C. difficile* infections (CDI) are the most frequently reported nosocomial infections that represent growing and an expensive health care burden, especially in elderly and hospitalized patients [4].

*C. perfringens* has been traditionally associated with enteric infection of pets and farm animals including goats, sheep and poultry [5]. In addition, *C. perfringens* type A food poisoning represents the second most common food-borne disease in developed countries [6]. *C. perfringens* enterotoxin causes food- and nonfood-borne human gastrointestinal diseases – enterotoxaemia in which toxins get into the circulation and damage various organs, including brain [2].

The current treatment of CDI is based on the use of antibiotics metronidazole and vancomycin, although the high rates of recurrence occur. Hence, novel therapeutic strategies are evaluated, faecal microbiota transplantation, new antibiotics, probiotics, bacteriocins, and bacteriophages, in order to neutralize the *C. difficile* effects in humans [7]. Moreover, due to the European ban on the use of antibiotics in livestock in the 2006, numerous studies have been performed in order to establish alternative strategies for animal diseases prevention. A particular attention has been paid on the use of probiotics defined as “live microorganisms that, when administered in adequate amounts, confer a health benefit on the host” [8, 9].

The aim of this study was to evaluate in vitro and in vivo probiotic potential of novel designed probiotic culture consisting of *Lactobacillus helveticus* BGRA43, *Lactobacillus fermentum* BGHI14 and *Streptococcus thermophilus* BGVLJ1-44 for possible use in prevention and treatment of infections caused by *Clostridium difficile* and *C. perfringens* in humans and animals. The strain BGRA43 exhibits wide-range antimicrobial activity against various pathogenic bacteria including, among others, *Yersinia enterocolitica, Shigella sonnei, Shigella flexneri, Shigella dysenteriae* and *Clostridium sporogenes* [10]*.* Bioactive peptides obtained after proteolytic activity of BGRA43 on milk proteins have potent immunomodulatory activity by suppressing the production of proinflammatory cytokines IL-6 and TNF-α [11]. The strain *L. fermentum* BGHI14 was successfully used in prevention and treatment of experimental TNBS-induced colitis in rats, where the non-invasive and safe nature of the strain was determined. The strain BGHI14 boosted mucosal defence systems and induced elevated production of protective cytokines IL-1β and TNF-α in the treated rats [12]. The exopolisaccharide-producing strain *S. thermophilus* BGVLJ1-44 was chosen due to its good technological properties with the aim to use the novel probiotic as starter culture for preparation of fermented dairy functional foods and feeds. According to our knowledge, this is the first publication reporting the successful elimination of *C. perfringens* in goats.

## Methods

### Bacterial strains and the growth conditions

Natural dairy isolate *S. thermophilus* BGVLJ1-44 and human intestinal isolates *L. fermentum* BGHI14 [12] and *L. helveticus* BGRA43 [10] from laboratory collection were used. All strains were deposited in Belgian Coordinated Collection of Microorganisms (BCCM), Laboratory for Microbiology, University of Ghent, Belgium, K.L. Ledegancstraat 35, Ghent, Belgum. The strain *Streptococcus thermophilus* BGVLJ1-44 was deposited under LMG P-28585 number, the strain *Lactobacillus fermentum* BGHI14 under LMG P-28583 number, and the strain *Lactobacillus helveticus* BGRA43 has a deposit number LMG P-24226.

BGVLJ1-44 was grown in liquid or solid M17 medium (Merck GmbH, Darmstadt, Germany), supplemented with 0.5% glucose (GM17), while BGHI14 and BGRA43 strains were grown in liquid and solid MRS medium (Merck, GmbH). All strains were grown at 37 °C in anaerobic conditions (Anaerocult A, Merck, GmbH). *C. difficile* was grown in sulphite agar (Torlak, Belgrade, Serbia). The enumeration of *C. perfringens* was performed on TSC agar [13].

### Antibiotic susceptibility testing

Minimal inhibitory concentrations (MICs) were determined by microdilution testing following the European Food Safety Authority resomendations [14]. Antibiotic susceptibility of *L. helveticus* BGRA43 was tested against ampicillin (1 μg/ml), erythromycin (1 μg/ml), tetracycline (4 μg/ml), chloramphenicol (4 μg/ml), and gentamicin (16 μg/ml), susceptibility of *L. fermentum* BGHI14 was tested against ampicillin (2 μg/ml), erythromycin (1 μg/ml), tetracycline (8 μg/ml), chloramphenicol (4 μg/ml), and gentamicin (16 μg/ml) and susceptibility of *S. thermophilus* BGVLJ1**-**44 was tested against ampicillin (2 μg/ml), vancomycin (4 μg/ml), erythromycin (2 μg/ml), tetracycline (4 μg/ml), chloramphenicol (4 μg/ml), and gentamicin (32 μg/ml). Experiments were done in triplicate. The cell density was obtained after 24 h incubation at 37 °C, by measuring of absorbance at 595 nm using Plate Reader Infinite 200 pro. MICs values were determined as the lowest concentration of antibiotic that inhibit visible growth of bacteria.

### Antimicrobial activity test

Antimicrobial activity of the strains and probiotic combination on *C. difficile* was tested as described previously [15]. Briefly, the glass tubes were filled with overnight culture of *C. difficile* (1 ml; human clinical isolate) as indicator strain, followed by 5 ml of soft agar of Clostridium difficile agar base medium, and on top of it with 5 ml of the soft GM17 containing 200 μl of overnight culture of *Streptococcus thermophilus* LMG P-28585, or MRS with 200 μl of overnight cultures of *Lactobacillus helveticus* LMG P-24226 or *Lactobacillus fermentum* LMG P-28583 or 200 μl of mixed probiotic culture. Bacteria were grown at 37 °C in anaerobic conditions (Anaerocult A, Merck, GmbH). Production of gas by *Clostridium sporogenes* and *Clostridium difficile*, manifested in the form of cracking agar, represents the signal for the growth of *Clostridium sporogenes* and *Clostridium difficile* and absence of antimicrobial activity. The antimicrobial activity of probiotic strains on *Clostridium sporogenes* and *Clostridium difficile* is scored as the absence of gas production, i.e. agar remains intact.

### Maintenance of Caco-2 cells

Caco-2 cell line was purchased from the European Collection of Cell Cultures (ECACC No. 86010202). The culture and maintenance of the cell line was carried out as previously described [16] using Dulbecco’s modified Eagle’s medium (Thermo Fisher Scientific, USA) with 10% foetal bovine serum (FBS) (Thermo Fisher Scientific, USA), 100 U/ml of penicillin, 100 μg/ml of streptomycin and 4 mmol/L glutamine (Sigma, St. Louis, MO, USA). For the experiments 2 × 10^5^ Caco-2 cells per well (passage 15–30) were seeded in 24-well plates (Corning Costar, Sigma-Aldrich, Munich, Germany), cultivated at 37 °C in a 5% CO_2_-humidified incubator and collected at 60% confluence.

### Cytotoxicity assay

Caco-2 cells are widely used experimental model of intestinal epithelial cells (EIC), which form a physical and biochemical barrier to commensal and pathogenic microorganisms. So, we used these cells for in vitro cytotoxicity analysis. Caco-2 cells were seeded in 96-well microplate at a concentration of 1.2 × 10^4^ cells per well in growth medium. After 24 h of post seeding (40–60% confluency) bacterial suspensions were added at a ratio of about 10:1 (bacteria:eukaryotic cells). Cytotoxicity was determined using the Cytotoxicity Detection Kit (Roche Applied Science; Indianapolis, IN). Low controls consisted of supernatant from non-stressed Caco-2 cells with no exposure to bacteria. High controls were from cells treated with 1% Triton X-100 for 1 min. All culture supernatants were collected and centrifuged at 800×g for 5 min to remove bacterial and eukaryotic cells. An aliquot (100 μl) of each sample was immediately dispensed in triplicate wells of a 96 well plate and lactate dehydrogenase (LDH) activity was determined according to manufacturer’s protocol.

### Determination of the effect of bacterial strains on cytokine production by Caco-2 cell line

The cellular monolayer was carefully washed twice with PBS and treated with 1 × 10^7^ CFU/ml UV-irradiated bacteria of each tested strain or combination of three strains for 24 h. Bacterial suspensions were added at a ratio of about 10:1 (bacteria:eukaryotic cells). After 24 h of Caco-2 cells’ exposure to bacterial suspensions, culture supernatants were collected and centrifuged at 800×g for 5 min to remove bacterial and eukaryotic cells. By using commercially available ELISA kits, concentrations of IL-1β, IL-6, TGF-β and IL-27 (DuoSet ELISA kit, R&D Systems, Minneapolis, USA) were measured.

### The proliferation of GALT cells culture

The effect of UV-irradiated or live strains BGVLJ1-44, BGHI14 and BGRA43 and the probiotic combination prepared in milk on proliferation of rats’ GALT-isolated lymphocytes was monitored. Isolation of GALT cells was performed as previously described [17]. Essentialy, bacterial cells were inactivated by UV light for three cycles of 30 min each. After UV treatment plate counting was carried out to confirm the absence of live bacteria that might be able to recover in the proper medium. UV-inactivated bacteria were then divided in single aliquots, frozen in liquid nitrogen and stored at −80 °C until use. This study was approved by the Animal Experimentation Ethical Committee of the Faculty of Pharmacy, University of Belgrade (Serbia), in strict adherence to international directives. A total number of three Wistar rats (healthy female adults 6 weeks old) were purchased from the Farm of the Military Medical Academy, Belgrade (Serbia). For the experiments each animal was anaesthetized with CO_2_ and, once assured of the loss of corneal reflex, its intestine was excised from the jejunum to the ileocaecal junction. The whole small intestine was placed in cold Hank’s balanced salt solution (HBSS without calcium and magnesium ions, prepared according to the formulation of Gibco, Invitrogen, Carlsbad, CA, USA) and kept at 4 °C until processing. Finally, the animals were sacrificed using the increase of CO_2_ concentration. The isolation of lymphocytes from GALT (Peyer’s Patches lymphocytes and intestinal epithelium lymphocytes) was carried out as previously described [17].

To quantify the response of GALT to the different factors tested, 2 × 10^5^ lymphocyte cells were incubated with UV-inactivated bacteria (at a ratio of 1:5) for 4 days in complete RPMI medium with antibiotics at 37 °C and 5% CO_2_. All cultures were performed in triplicate in 96-well round-bottom microtiter plates. After 4 days of incubation, the proliferation of GALT cells was performed by using the ELISA-BrdU proliferation assay kit (GE Healthcare Ltd. Buckinghamshire, UK) following the manufacturer’s instructions. Results were compared to a negative control (lymphocytes growing in complete RPMI medium with antibiotics).

### Toxicology analysis

Acute and sub-acute toxicology analysis was performed on 12 mice (six experimental and six control animals) of NMRI HAN in accordance to the European convention for the protection of vertebrate animals used for experimental and other scientific purposes (Directive 2010/63/EU). Female and male 5 weeks old mice from Department for Biomedical Investigation, Galenika A.D., Belgrade, Serbia were used. All experimental procedures and protocols conformed to the International Guiding Principles for Biomedical Research Involving Animal. The experiments were approved by Ethical Committee of Galenika a.d., Serbia No 04/15. For acute toxicology analysis the mice were treated with a single dose of each bacterial strain or probiotic combination (10^8^ CFU/ml; 0.5 ml/mice), while for sub-acute toxicology analysis mice were treated with a probiotic combination (10^9^ CFU/ml; 0.5 ml/mice) for 14 days. The signs of toxic activity were followed twice a day during the first 24 h, and once a day during next 14 days. Body mass was measured twice a week. After 14 days from the beginning of the treatment, animals were sacrificed and the organs (liver, spleen, kidneys, gut, intestines, lungs, and heart) and tissue were examined macroscopically.

### Field trial: in vivo testing the antimicrobial effect of the probiotic combination

The effect of the mixed probiotic combination on the goats spontaneously infected with *C. perfringens* was tested. The study was conducted on commercial medium-size farm in Serbia, where several cases of *C. perfringens* infection were previously detected and resulted with lethal outcome. Hence, 10 female Saanen goats of an average age of 9 months were selected for the experiment on the basis of microbiological analysis of faecal samples confirming that the goats were carriers of *C. perfringens* (10^2^–10^3^ CFU/g). The experiments were performed in accordance to the European convention for the protection of vertebrate animals used for experimental and other scientific purposes (Directive 2010/63/EU) and approved by Ethical Committee of Faculty of Biology, University of Belgrade, Serbia; No EK-BF-2013/09). The goats were housed in individual pens (2.00 m × 2.00 m). The goats were treated with 50 ml of mixed probiotic culture for 10 days. The 10 bottles of mixed probiotic cultures were prepared at first day of the experiment by mixing the overnight cultures as follows: 150 ml of *S. thermophilus* BGVLJ1-44 (3.34 × 10^7^ CFU/ml), 150 ml of *L. helveticus* BGRA43 (5.6 × 10^8^ CFU/ml) and 200 ml of *L. fermentum* BGHI14 (4.1 × 10^8^ CFU/ml), respectively. One bottle was used for the first day of experiment and the nine bottles were kept at 4 °C for the next 9 days. Total number of viable bacteria (CFU) in the mixed probiotic cultures during the storage remained unchanged.

The faecal samples were collected before and after the treatment for microbiological analysis. Samples (1 g) were homogenized in 9 ml of sterile saline in a stomacher (AES; Comburg, France) for 90 s. Serial dilutions (10-fold) were made in sterile saline and plated on GM17 (Merck) for enumeration of lactococci/streptococci, MRS agar (Merck) for enumeration of lactobacilli, VRBD agar (Merck) for enumeration of *Enterobacteriaceae*, Luria agar (LA) for enumeration of *Bacillus* sp. and TSC agar [13] for enumeration of *C. perfringens* (black colonies) and other spore forming bacteria (white colonies). The lactococci and lactobacilli were grown at 37 °C for 48 h, *Enterobacteriaceae* and *Bacillus* sp. at 37 °C for 24 h, and *C. perfringens* at 37 °C for 48 h.

### Statistical analysis

All values obtained in the experiments were analysed with Kolmogorov-Smirnov test suggesting that the data followed Gaussian distribution. Differences between treatments and control culture were examined for significance by Student’s t-test. *P* > 0.05 was considered to be statistically insignificant.

## Results

### Evaluation of antimicrobial activity of probiotic against *C. difficile*

**Fig. 1:**
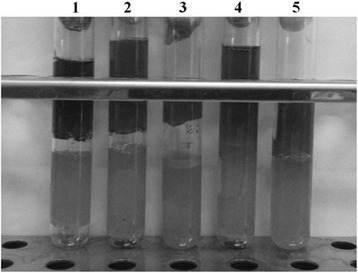
Antimicrobial activity of individual strains and probiotic combination on *Clostridium difficile*. 1. Control; 2. *C. difficile* + *S. thermophilus* BGVLJ1-44; 3. *C. difficile* + *L. fermentum* BGHI14; 4. *C. difficile* + *L. helveticus* BGRA43; 5. *C. difficile* + probiotic combination

The in vitro antimicrobial analysis revealed that the single culture of BGRA43 and the probiotic combination successfully suppressed the growth of *C. difficile* – there was no gas production or cracking of the sulphite agar. The single culture of BGVLJ1-44 partially suppressed the growth of *C. difficile* – slight gas production was observed on the bottom of the tube and the sulphite agar was partially cracked. Interestingly, in the presence of the single culture of BGHI14 there was no gas production observed on the bottom of the tube, while the sulphite agar was cracked, possibly due to the gas production by the heterofermentative *L. fermentum* BGHI14 strain (Fig. 1).

### Determination of the safety status of the probiotic

With the aim of safe use of the probiotic in humans and animals, antibiotic susceptibility of the probiotic strains was analyzed. The results showed that the strains BGRA43, BGHI14 and BGVLJ1-44 are sensitive to the recommended levels of antibiotics [14]. In this way, the basic prerequisite for the use of the strains as probiotic is met.

**Fig. 2:**
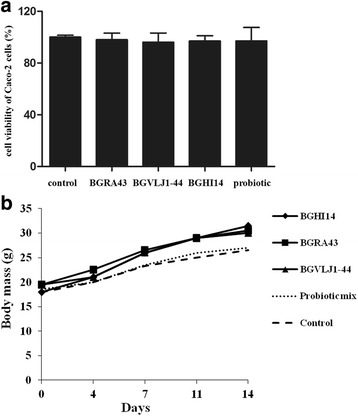
**a** Cytotoxic effect of individual strains and probiotic combination on Caco-2 cells. The values given in the graph represent mean values of three independent experiments ± standard deviation and are expressed in percentages. All experiments were done in triplicates. **b** Toxicology analysis of individual strains and probiotic combination in mice. The body weights of the laboratory animals fed with individual strains and probiotic combination in mice for 14 days is presented

In addition, we exposed Caco-2 cells to UV-irradiated bacterial strains and their combination to determine their possible cytotoxic effect. The results showed that neither of the tested strains nor the probiotic combination induced the elevated release of LDH from Caco-2 cells during 24 h cultivation indicating that tested bacterial strains BGRA43, BGHI14 and BGVLJ1-44 did not exhibit cytotoxic effect on the used epithelial cells (Fig. 2a).

In addition, the results of in vivo acute and sub-acute toxicology analysis of single bacterial strains and probiotic combination revealed that none of the strains, or a probiotic combination exhibited toxic effects. The animals behaved normally as expected for their species, sex, age and environment. There were no signs of neurological disturbances. Hygienic behaviour of animals was normal. Their eyes were bright and clean, the nasal and other natural openings were clean. The body weight was measured twice a week during the experimental period and the results were in accordance to the results obtained for the control animals (Fig. 2b). The experimental animals did not show eating disorders. During the experimental period none of animals died. Macroscopic examination of organs and tissues did not determine changes in any of the tested animals.

### Characterization of immunomodulatory properties of the probiotic

In addition to demonstrated antimicrobial activity possible immunomodulatory activity of the tested bacterial strains was followed in order to explore the other possible mechanisms by which the bacterial strains could prevent or control pathogens.

**Fig. 3:**
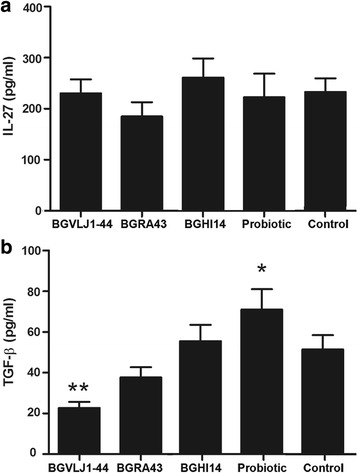
**a** and **b** Production of IL27 and TGFβ, respectively, by Caco-2 cells in the presence of *L. helveticus* BGRA43, *L. fermentum* BGHI14, and *S. thermophilus* BGVLJ1-44 and probiotic combination treated with UV. The values given in the graph represent mean values of three independent experiments ± standard deviation. All experiments were done in triplicates. Statistically significant differences between treatments and control are marked with asterisks (*p < 0.05, **p < 0.01)

**The production of IL-1β, IL-6, TGF-β and IL-27 cytokines by Caco-2 cells** in the presence of UV-inactivated bacterial cells of the strains BGRA43, BGHI14, BGVLJ1-44 and their combination was followed. The obtained results showed that Caco-2 cells did not produce proinflammatory cytokines IL-6 and IL-1β, neither spontaneously or induced by the presence of bacterial suspensions (single or mixed). On the other hand, Caco-2 cells spontaneously produced IL-27 (Fig. 3a), although there were no significant changes in the IL-27 production in the presence of the bacterial suspensions. Finally, Caco-2 cells produced TGF-β spontaneously (Fig. 3b) and in the presence of the strains BGHI14 and BGRA43 the production was not significantly modified. Interestingly, the strain BGVLJ1-44 significantly (*p* < 0.01) diminished the production of TGF-β, while probiotic combination induced significantly (*p* < 0.05) elevated production of this cytokine.

**Fig. 4:**
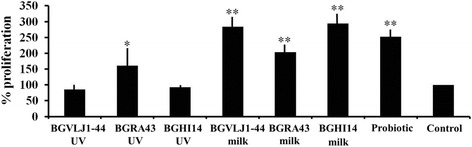
Proliferation of rat’s GALT-isolated lymphoide cells in the presence of UV-irradiated and live strains *S. thermophilus* BGVLJ1-44, *L. fermentum* BGHI14 and *L. helveticus* BGRA43, as well as probiotic combination prepared in milk. The values given in the graph represent the mean values of three independent experiments ± standard deviation and are expressed in percentages. All experiments were done in quadruplicates. Statistically significant differences between treatments and control are marked with asterisks (*p < 0.05, **p < 0.01)

**The proliferation of GALT-isolated lymphocytes** in the presence of UV-irradiated and live cells of BGRA43, BGHI14, BGVLJ1-44 and the mixed probiotic culture was followed during 3 days of co-incubation. The results are expressed as a percentage relative to the cells incubated in RPMI medium alone (Fig. 4). The obtained results showed that among UV-irradiated cells only the strain BGRA43 induced significantly (*p* < 0.05) higher proliferation of GALT-isolated lymphocytes compared to the control. Interestingly, the proliferation of GALT-isolated lymphocytes in the presence of all strains and the probiotic combination was significantly (*p* < 0.01) higher compared to control. The results indicate that live cells might have stronger stimulatory effect on the proliferation of GALT-isolated lymphocytes compared to UV-irradiated cells (Fig. 4).

### In vivo antimicrobial activity of probiotic on goats spontaneously infected by *C. perfringens*

**Fig. 5:**
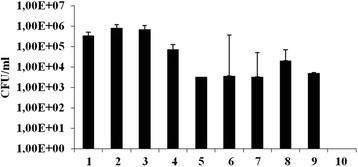
Enumeration of various bacterial groups before (1, 3, 5, 7, 9) and after (2, 4, 6, 8, 10) the treatment of goats by probiotic combination in the field trial. 1–2. Lactococci; 3–4. Lactobacilli; 5–6. *Bacillus* sp.; 7–8. Other spore forming bacteria; 9–10. *Clostridium perfringens*. The values given in the graph represent the mean values of four measurements ± standard deviation and are expressed in percentages

In order to determine potential protective and therapeutic effect of the probiotic on farm animals, the field trial was performed including 10 goats spontaneously infected by *C. perfringens* determined by local veterinary authority. Enumeration of *C. perfringens* showed that there was 10^2^–10^3^ CFU/g of faecal sample at the beginning of the experiment). Importantly, the results showed that the probiotic culture reduced the number of *C. perfringens* in faecal samples collected from goats after the probiotic treatment, below the detection level (Fig. 5). The general health of the tested goats was improved. In addition, no significant effect of the probiotic on other tested bacterial groups was observed (Fig. 5). Interestingly, moulds were noticed only in faecal samples collected before the probiotic treatment (data not shown).

## Discussion

The probiotic antagonistic activity against pathogens is a strain-dependent and could be achieved by diverse mechanisms, such as production of antimicrobial substances, reinforcement of the gut epithelium, colonization competition, and/or stimulation of the innate immune response [18].

Vast number of studies performed to evaluate antimicrobial activities of different probiotic strains and natural isolates revealed the direct antimicrobial effects against *C. difficile* and *C. perfringens* [8, 19]. There are strong evidences that probiotics *L. rhamnosus* GG and *Saccharomyces boulardii* have been efficient in prevention of *C. difficile* associated diarrhoea in combination with antibiotic therapy [20–23]. However, there are no sufficient evidences to support the use of the probiotics alone in treatment of *C. difficile* colitis [21]. Hence, the aim of this study was to evaluate antimicrobial activities of the strains BGRA43, BGHI14 and BGVLJ1-44 against *C. difficile*. Broad antimicrobial spectrum of the strain BGRA43 was previously reported, including the activity against *C. sporogenes*, although the exact nature of the antimicrobial compound(s) remains unknown [10]*.* Our results revealed that all three tested strains produce antimicrobial substance(s) active against *C. difficile*. Apparently, the strain BGRA43 had the most influence on antimicrobial activity of the probiotic combination.

Though, before the use of probiotics in humans and animals, several criteria need to be considered in order to confirm the safety of bacterial strains. Ideally, probiotic strains must be sensitive to relevant clinical antibiotics and lack the virulence genes [8]. Lactic acid bacteria are denoted as GRAS in healthy populations. In addition, our previous results revealed that bacterial strains used in this study do not carry antibiotic resistance genes [10, 12]. Nevertheless, healthy populations generally do not belong to the risk group for *C. difficile* infection. *C. difficile* risk factors mainly include increased age, severe underlying illnesses and long hospitalization; hence the risks of probiotic administration in such groups must be considered [21]. Therefore, the safe use of the strains BGRA43, BGHI14 and BGVLJ1-44 and the probiotic combination was evaluated. Caco-2 cells are widely used experimental model of EIC, which form a physical and biochemical barrier to commensal and pathogenic microorganisms. The obtained results showed that probiotic strains and their combination had no cytotoxic effects on Caco-2 cells, suggested that the intestinal epithelial barrier function of Caco-2 cells is maintained in the presence of probiotic. In addition, the results of toxicology analysis indicated that the strains, as well as probiotic combination, could be safely used both in humans and animals.

*C. difficile* infection is often the result of long-term antibiotic use and occurs when the protective barrier of the gut, provided by commensal microbiota, is disrupted. Finally, pathogenic bacteria and their toxins cause mucosal damage and inflammation, followed by diarrhoea. Hence, gut epithelium is the initial site of the interaction of commensal and pathogenic bacteria with the host. One of the important features of probiotic is to colonize gut, stabilize the permeability of IEC and inhibit the adherence of pathogens [9]. The results of our previous work, where it was deduced that the strain BGRA43 has significantly better adhesion trait than *L. rhamnosus* GG reference strain [10].

In addition, the immunomodulatory effects of tested probiotic strains on Caco-2 cells were explored. It is well recognised that IEC produce different immunomodulatory molecules important for maintenance of steady-state condition and direction of appropriate innate and adaptive immune response [24]. In line with this, the obtained results showed that the probiotic did not induce the production of pro-inflammatory cytokines (IL-1β, IL-6), but stimulated the production of TGF-β by Caco-2 cells. TGF-β produced by IECs in the response to commensal stimulation has indispensable role in the regulation of the development of antigen presenting cells with tolerogenic properties, such as tolerogenic dendritic cells [25, 26]. Subsequently, tolerogenic dendritic cells promote further the intestinal tolerance by inducing the differentiation of regulatory T cells and the maturation of IgA-secreting plasma cells through the secretion of IL-10, TGF-β and retinoic acid [27, 28]. Walia and co-authors [29] showed that TGF-β had direct protective effect on IEC by down-regulating IL-6 signalling in IEC. IL-6 signalling is important for host response to pathogen, but the over-expression of this cytokine is one of the major factors involved in the pathogenesis of different inflammatory diseases such as inflammatory bowel disease [30, 31]. Considering such an important immunoregulatory role of TGF-β, the ability of probiotic to stimulate the production of TGF-β by Caco-2 cells in the absence of pathogen could contribute to the maintenance of the intestinal epithelial barrier in steady-state condition. This is in accordance with literature where the stimulation of TGF-β production was correlated with potential protective effect of various probiotic strains in different inflammatory condition [32, 33]. In addition, the role of IL-27 as a mediator of intestinal epithelial barrier protection was characterized [34]. In line with this, we showed that Caco-2 cells produce IL-27 spontaneously and that this activity is maintained in the presence of probiotic strains.

On the other side, infection with pathogens such as *C. difficile* [35] contributes to the deregulation of continuous physical barrier leading to IEC cytoskeletal rearrangements and disrupture of tight junctions. The loss of paracellular barrier functions increases the barrier permeability and translocation of luminal bacteria. In such conditions, applied probiotics could be translocated across the intestinal epithelial barrier and directly affect underlying intestinal immune cells. Considering this, we further analysed the effect of live and UV-irradiated bacterial strains and their combination on proliferation of GALT, the dominant intestinal immune tissue. The results indicated potential immunostimulatory role of the live strains and the probiotic combination in the case of disrupted epithelial barrier, although the direct immunomodulatory role of the probiotic should be further evaluated.

Finally, several studies indicated that probiotic bacteria could be potential alternatives to the use of antibiotics in prevention of *C. perfringens*-induced necrotic enteritis in broiler chickens [8]. However, Schoster and co-authors [36] reported that a probiotic treatment of foals by mixed probiotic culture, consisting of four *Lactobacillu*s species and one *Bifidobacterium animalis lactis*, did not exhibit beneficial effect on *C. difficile* caused diarrhoea, even the potential adverse effects were determined. The authors discussed the possibility of inadequate choice of the strains or dose, despite their probiotic activity determined in vitro. Thus, one of the aims of this study was to test the efficacy of the probiotic culture in field trial*.* Interestingly, the obtained results showed that the probiotic was successful in eliminating the *C. difficile* in goats below the detection level. However, it is worth noting that the number of *C. difficile* viable cells before the probiotic treatment was quite low (10^2^–10^3^ CFU/g), pointing that the goats were just carriers of the infectious agent, raising questions of potential efficacy of the probiotic in the pick of disease. Nevertheless, the results of this study suggest that the novel probiotic could be potentially used in prevention of the infection.

## Conclusions

The results of this study indicate that the novel designed probiotic culture could be potentially used as a biological agent in prevention of *C. perfringens* infection in animals, as alternative to antibiotics. Additionally, the probiotic was proven to have strong antimicrobial effect against *C. difficile* in vitro, and exhibits protective immunomodulatory effect. However, the safety and health promoting efficacy of the probiotic against *C. difficile* infection in humans need to be proven in human clinical trials.

## Abbreviations

CFU: Colony forming unit
DCs: Dendritic cells
FBS: Foetal bovine serum
GALT: Gut associated lymphoid tissue
GRAS: Generally regarded as safe
IEC: Intestinal epithelial cells
IL: Interleukin
LDH: Lactate dehydrogenase
TGFβ: Transforming growth factor β
TNBS: 2,4,6-trinitrobenzenesulfonic acid
TNF-α: Tumor necrosis factor α

## References

[1] Janezic S, Zidaric V, Pardon B, Indra A, Kokotovic B, Blanco JL, Seyboldt C, Diaz CR, Poxton IR, Perreten V, Drigo I, Jiraskova A, Ocepek M, Weese JS, Songer JG, Wilcox MH, Rupnik M (2014). International *Clostridium difficile* animal strain collection and large diversity of animal associated strains. BMC Microbiol.

[2] Uzal FA, Freedman JC, Shrestha A, Theoret JR, Garcia J, Awad MM, Adams V, Moore RJ, Rood JI, McClane BA (2014). Towards an understanding of the role of *Clostridium perfringens* toxins in human and animal disease. Future Microbiol.

[3] Seekatz AM, Young VB (2014). *Clostridium difficile* and the microbiota. J Clin Invest.

[4] Kujawa-Szewieczek A, Adamczak M, Kwiecien K, Dudzicz S, Gazda M, Wiecek A (2015). The effect of *Lactobacillus plantarum* 299v on the incidence of *Clostridium difficile* infection in high risk patients treated with antibiotics. Nutrients.

[5] Silva RO, Lobato FC (2015). *Clostridium perfringens*: A review of enteric diseases in dogs, cats and wild animals. Anaerobe.

[6] McClane BA, Robertson SL, Li J, Doyle MP, Buchanan RL (2013). *Clostridium perfringens*. Food microbiology: fundamentals and frontiers.

[7] Valdes-Varela L, Alonso-Guervos M, Garcia-Suarez O, Gueimonde M, Ruas-Madiedo P (2016). Screening of bifidobacteria and lactobacilli able to antagonize the cytotoxic effect of *Clostridium difficile* upon intestinal epithelial HT29 monolayer. Front Microbiol.

[8] Caly DL, D’Inca R, Auclair E, Drider D (2015). Alternatives to antibiotics to prevent necrotic enteritis in broiler chickens: A microbiologist’s perspective. Front Microbiol.

[9] Joint Food and Agriculture Organization/World Health Organization (2016). FAO–WHO. Probiotics in food: health and nutritional properties and guidelines for evaluation.

[10] Strahinic I, Lozo J, Terzic-Vidojevic A, Fira D, Kojic M, Golic N, Begovic J, Topisirovic L (2013). Technological and probiotic potential of BGRA43 a natural isolate of *Lactobacillus helveticus*. Front Microbiol.

[11] Tompa G, Laine A, Pihlanto A, Korhonen H, Rogelj I, Marnila P (2011). Chemiluminescence of non-differentiated THP-1 promonocytes: developing an assay for screening anti-inflammatory milk proteins and peptides. Luminescence.

[12] Lukic J, Strahinic I, Milenkovic M, Golic N, Kojic M, Topisirovic L, Begovic J (2013). Interaction of *Lactobacillus fermentum* BGHI14 with rat colonic mucosa: implications for colitis induction. Appl Environ Microbiol.

[13] Adams BW, Mead GC (1980). Comparison of media and methods for counting *Clostridium perfringens* in poultry meat and further-processed products. J Hyg.

[14] European Food Safety Authority (EFSA). Guidance on the assessment of bacterial susceptibility to antimicrobials of human and veterinary importance. EFSA panel on additives and products or substances used in animal feed (FEEDAP). EFSA Journal. 2012;10:2740.

[15] Banina A, Vukasinovic M, Brankovic S, Fira D, Kojic M, Topisirovic L (1998). Characterization of natural isolate *Lactobacillus acidophilus* BGRA43 useful for acidophilus milk production. J Appl Microbiol.

[16] Nikolic M, Lopez P, Strahinic I, Suarez A, Kojic M, Fernandez-Garcia M, Topisirovic L, Golic N, Ruas-Madiedo P (2012). Characterisation of the exopolysaccharide (EPS)-producing *Lactobacillus paraplantarum* BGCG11 and its non-EPS producing derivative strains as potential probiotics. Int J Food Microbiol.

[17] Hidalgo-Cantabrana C, Nikolic M, López P, Suárez A, Miljkovic M, Kojic M, Margolles A, Golic N, Ruas-Madiedo P (2014). Exopolysaccharide-producing *Bifidobacterium animalis* subsp. *lactis* strains and their polymers elicit different responses on immune cells from blood and gut associated lymphoid tissue. Anaerobe.

[18] Boirivant M, Strober W (2007). The mechanism of action of probiotics. Curr Opin Gastroenterol.

[19] Ollech JE, Shen NT, Crawford CV, Ringel Y (2016). Use of probiotics in prevention and treatment of patients with *Clostridium difficile* infection. Best Pract Res Clin Gastroenterol.

[20] Hickson M, D’Souza AL, Muthu N, Rogers TR, Want S, Rajkumar C, Bulpitt CJ (2007). Use of probiotic *Lactobacillus* preparation to prevent diarrhoea associated with antibiotics: randomised double blind placebo controlled trial. BMJ.

[21] Hickson M (2011). Probiotics in the prevention of antibiotic-associated diarrhoea and *Clostridium difficile* infection. Therap Adv Gastroenterol.

[22] Plummer S, Weaver MA, Harris JC, Dee P, Hunter J (2004). *Clostridium difficile* pilot study: effects of probiotic supplementation on the incidence of *C. difficile* diarrhoea. Int Microbiol.

[23] Surawicz CM, McFarland LV, Greenberg RN, Rubin M, Fekety R, Mulligan ME, Garcia RJ, Brandmarker S, Bowen K, Borjal D, Elmer GW (2000). The search for a better treatment for recurrent *Clostridium difficile* disease: use of high-dose vancomycin combined with *Saccharomyces boulardii*. Clin Infect Dis.

[24] Peterson LW, Artis D (2014). Intestinal epithelial cells: regulators of barrier function and immune homeostasis. Nat Rev Immunol.

[25] Zeuthen LH, Fink LN, Frokiaer H (2008). Epithelial cells prime the immune response to an array of gut-derived commensals towards a tolerogenic phenotype through distinct actions of thymic stromal lymphopoietin and transforming growth factor-beta. Immunology.

[26] Rimoldi M, Chieppa M, Salucci V, Avogadri F, Sonzogni A, Sampietro GM, Nespoli A, Viale G, Allavena P, Rescigno M (2005). Intestinal immune homeostasis is regulated by the crosstalk between epithelial cells and dendritic cells. Nat Immunol.

[27] Coombes JL, Siddiqui KR, Arancibia-Carcamo CV, Hall J, Sun CM, Belkaid Y, Powrie F (2007). A functionally specialized population of mucosal CD103+ DCs induces Foxp3+ regulatory T cells via a TGF-beta and retinoic acid-dependent mechanism. J Exp Med.

[28] Macpherson AJ, Uhr T (2004). Induction of protective IgA by intestinal dendritic cells carrying commensal bacteria. Science.

[29] Walia B, Wang L, Merlin D, Sitaraman SV (2003). TGF-beta down-regulates IL-6 signaling in intestinal epithelial cells: critical role of SMAD-2. FASEB J.

[30] Heinrich PC, Behrmann I, Haan S, Hermanns HM, Muller-Newen G, Schaper F (2003). Principles of interleukin (IL)-6-type cytokine signalling and its regulation. Biochem J.

[31] Reinisch W, Gasche C, Tillinger W, Wyatt J, Lichtenberger C, Willheim M, Dejaco C, Waldhör T, Bakos S, Vogelsang H, Gangl A, Lochs H (1999). Clinical relevance of serum interleukin-6 in Crohn’s disease: single point measurements, therapy monitoring, and prediction of clinical relapse. Am J Gastroentrol.

[32] Huang IF, Lin IC, Liu PF, Cheng MF, Liu YC, Hsieh YD, Chen JJ, Chen CL, Chang HW, Shu CW (2015). *Lactobacillus acidophilus* attenuates *Salmonella*-induced intestinal inflammation via TGF-beta signaling. BMC Microbiol.

[33] Barletta B, Rossi G, Schiavi E, Butteroni C, Corinti S, Boirivant M, Di Felice G (2013). Probiotic VSL#3-induced TGF-beta ameliorates food allergy inflammation in a mouse model of peanut sensitization through the induction of regulatory T cells in the gut mucosa. Mol Nutr Food Res.

[34] Diegelmann J, Olszak T, Goke B, Blumberg RS, Brand S (2012). A novel role for interleukin-27 (IL-27) as mediator of intestinal epithelial barrier protection mediated via differential signal transducer and activator of transcription (STAT) protein signaling and induction of antibacterial and anti-inflammatory proteins. J Biol Chem.

[35] Leslie JL, Huang S, Opp JS, Nagy MS, Kobayashi M, Young VB, Spence JR (2015). Persistence and toxin production by *Clostridium difficile* within human intestinal organoids result in disruption of epithelial paracellular barrier function. Infect Immun.

[36] Schoster A, Staempfli HR, Abrahams M, Jalali M, Weese JS, Guardabassi L (2015). Effect of a probiotic on prevention of diarrhea and *Clostridium difficile* and *Clostridium perfringens* shedding in foals. J Vet Intern Med.

